# Multiquadrant presentation of eruption sequestrum: a case report and literature review

**DOI:** 10.1186/s12903-025-06676-x

**Published:** 2025-09-03

**Authors:** Xinyi Lu, Leitao Li, Xili Qiu, Long Li, Binjie Liu, Ling Peng

**Affiliations:** Xiangya Stomatological Hospital and Xiangya School of Stomatology, Hunan Key Laboratory of Oral Health Research, Hunan Clinical Research Center of Oral Major Diseases and Oral Health, Academician Workstation for Oral-maxilofacial and Regenerative Medicine, Central South University, Changsha, 410008 China

**Keywords:** Tooth eruption, Molars, Bone tissue, Inflammation, Histopathology

## Abstract

**Background:**

Eruption sequestrum (ES) is an uncommon condition characterized by irregular calcified tissues on the occlusal surfaces of erupting permanent molars, primarily in children. Despite its rarity, ES has been linked to potential complications, including gingivitis, occlusal trauma, and delayed tooth eruption.

**Case presentation:**

This paper reports a multiquadrant ES case affecting the first molars of bilateral maxillary and mandibular arches in a 6-year-old pediatric patient. Intraoral examination revealed small, pale-yellow calcified tissues on the distal occlusal surfaces of the erupting first molars. Periapical radiographic evaluation revealed distinct radiopaque masses on the occlusal surfaces of the molars. Histopathologic examination identified the masses as nonviable compact bone tissue surrounded by chronic inflammation.

**Conclusions:**

This report enhances the understanding of tooth eruption disorders by documenting quadrant-wide ES, which ultimately provides insights into its diagnosis and treatment in pediatric patients.

## Introduction

Eruption sequestrum (ES) refers to a rare phenomenon characterized by irregular calcified tissue within the soft tissue overlying the occlusal surfaces of erupting permanent molars, more commonly observed above the central fossa in children [1]. Radiographically, ES appears as a small, irregular radiopaque mass separating from the tooth. Typically, ES is resorbed or exfoliated without causing symptoms and generally does not require specific intervention. However, in some cases, ES can induce mechanical irritation, occlusal trauma, or bacterial plaque accumulation, which may result in gingivitis, dental caries, or delayed tooth eruption, thereby requiring clinical intervention. This report details an ES case and its pathological findings involving bilateral maxillary and mandibular first molars in a 6-year-old girl.

## Case presentation

**Fig. 1:**
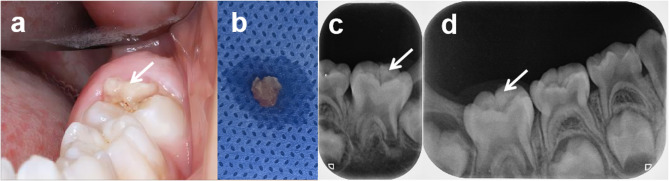
Initial findings. **a** An irregular calcified tissue observed on the distal occlusal surface of tooth 36. **b** The detached calcified tissue from tooth 36. **c** A radiopaque mass above the occlusal surface of tooth 36. **d** A radiopaque mass beneath a low-density shadow above the occlusal surface of tooth 46.

A 6-year-old girl presented to the Pediatric Dentistry Department of Xiangya Stomatological Hospital, Central South University, with a one-week history of discomfort from food getting stuck in the lower left back teeth, along with slight gum bleeding after eating. No treatment had been sought prior to the visit. The patient had no significant medical history and no relevant family history. Clinical examination revealed a partially erupted tooth 36 with mobile, pale-yellow, and irregularly shaped calcified tissue (5 mm×5 mm) on its distal occlusal surface, partially covered by an inflamed gingival tissue (Fig. 1a-b). Tooth 46 was unerupted with normal gingival tissue. Radiological evaluation using periapical radiographs showed irregular and radiopaque masses with enamel-like density over teeth 36 (Fig. 1c) and 46 (Fig. 1d).

**Fig. 2:**
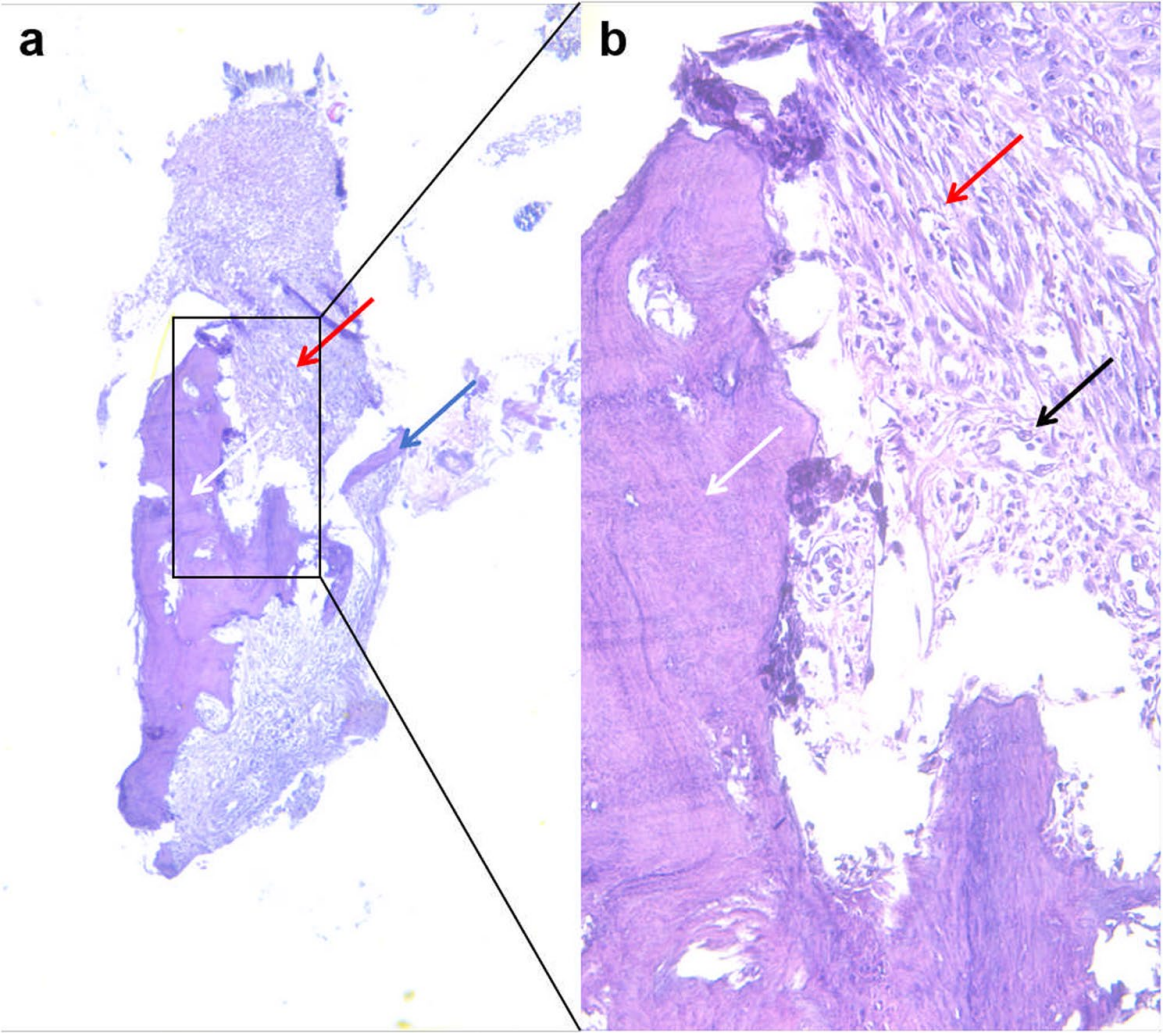
Histopathologic examination of ES (H&E stain). Bone tissue (white arrow), squamous epithelial tissue (blue arrow), fibrous tissue hyperplasia (red arrow), and interstitial inflammatory cell infiltration (black arrow) are identified. (a: 50×, b: 200×)

After obtaining informed consent from her guardian, local anesthesia (4% articaine) was administered, and the calcified tissue over tooth 36 was excised with a surgical blade, along with a small portion of the firmly adherent gingival tissue, to ensure complete removal of the lesion and to enable histopathological assessment of the gingiva-sequestrum interface. Histopathologic examination confirmed the presence of dense, nonviable compact bone tissue (Fig. 2a). Squamous epithelial tissue with organized cell alignment was observed, along with subepithelial fibrous tissue hyperplasia and inflammatory cell infiltration without detectable microbial colonization, consistent with chronic inflammation (Fig. 2b). Based on the patient’s clinical manifestations, radiographic findings, and histopathologic examination, the diagnosis was confirmed as eruption sequestrum.

**Fig. 3:**
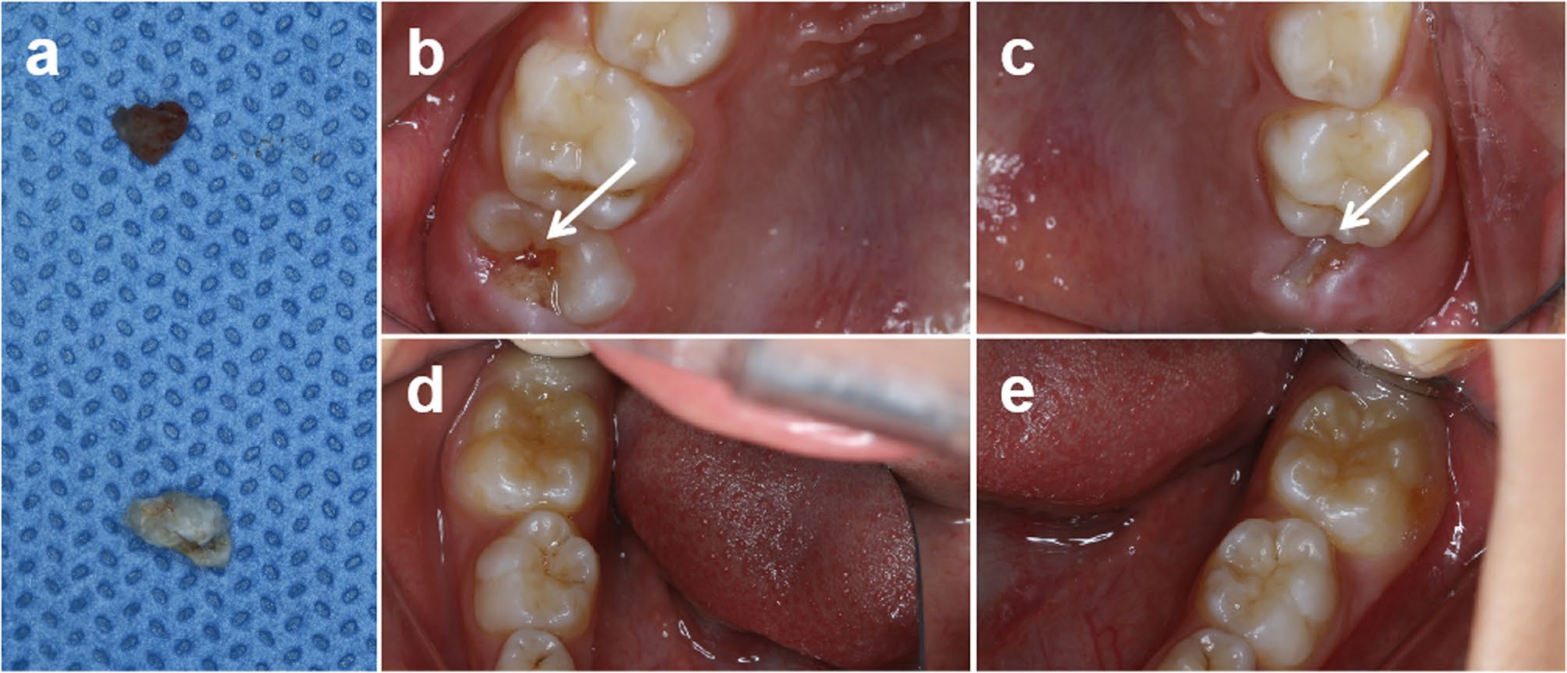
Follow-up findings. (**a**) The detached calcified mass from teeth 16 (upper) and 46 (lower). (**b**) An irregular calcified tissue over the occlusal surface of tooth 16. (**c**) Eruption of tooth 26. (**d**) Normally erupting tooth 46. (**e**) Normally erupting tooth 36. Note: Panels are arranged according to anatomical orientation

Postoperative instructions were given, and follow-up appointments were scheduled. One month post-procedure, spontaneous exfoliation of a calcified tissue was reported during the eruption of tooth 46 (Fig. 3a-lower). At the three-month follow-up, a similar mass was noted over tooth 16 (Fig. 3a-upper and b), which was partially erupted and associated with inflamed gingival tissue. The mass was removed afterward. During the same visit, clinical examination of the erupting tooth 26 revealed exposure of the mesio-palatal cusp, with a firm calcified mass detectable on the occlusal surface and subgingivally upon periodontal probing (Fig. 3c). The family opted for observational monitoring rather than intervention at that time, and further follow-up was discontinued. The teeth 36 and 46 erupted normally without any complications (Fig. 3d-e).

## Literature review

Eruption sequestrum, a rare dental anomaly, was initially described by Starkey and Shafer [1] in 1963. To date, a comprehensive review of the English literature has documented only 21 reported cases of ES [1–12]. Based on our analysis of these cases, ES predominantly occurs unilaterally (66.7%), in the mandible (76.2%), in both the mandible and maxilla (9.5%), on first molars (76.2%), and on second molars (23.8%), exhibiting minimal gender disparity (57.1% male versus 42.9% female) (Table 1).

**Table 1:** Statistical profile of 21 documented eruption sequestrum (ES) cases

Category			Unilateral						Bilateral						Total
			Maxilla		Mandible		Both		Maxilla		Mandible		Both		
			M6	M7	M6	M7	M6	M7	M6	M7	M6	M7	M6	M7	
Gender	Male	2^4,6^		/	4^1,2,4^	1^3^	/	/	/	/	1^4^	2^4,7^	1^5^	/	11
	Female	1^3^		/	4^1,10–12^	2^3,9^	/	/	/	/	2^8^	/	1^5^	/	10
Total			3	/	8	3	/	/	/	/	3	2	2	/	21

M6 first permanent molar, M7 second permanent molar

### Etiological controversies and emerging hypotheses

The pathogenesis of ES remains debated. Starkey and Shafer [1] originally hypothesized that cortical bone fragments trapped during eruption were the primary etiology. However, the clinical manifestations reported by Watkins [2] and Priddy et al. [5] in cases consistent with ES demonstrated that the calcified fragments were predominantly composed of dentin and cementum, rather than free bone tissue. Similarly, Onishi et al. [8] identified the presence of dentin, cementum, and pulp-like components within the hard tissue. The pulp-like connective tissue within the mass was richly vascularized, and the cells embedded in the osteodentin-like components remained active, suggesting that the calcified tissue was in a continuous developmental state. Based on these findings, Onishi et al. [8] proposed, for the first time, that ES might represent a calcifying hamartoma of mesenchymal origin. The histopathological analysis conducted by Roza et al. [12] further supports this perspective, defining ES as a hamartoma with limited developmental potential. Additionally, they noted chronic inflammatory infiltrates in the soft tissue. Integrating these observations with previous literature documenting chronic inflammation in the soft tissue, Roza et al. [12] suggested the term inflamed odontogenic hamartoma to more accurately describe ES. This nomenclature better encapsulates the pathological characteristics and developmental nature of the lesion.

### Histopathological and biochemical insights

Histologically, ES typically presents as non-viable, dense bone with empty lacunae lacking osteocytes, often accompanied by evidence of necrosis [1, 4, 7, 9–11]. Limited histopathological descriptions are available regarding the adjacent soft tissues in ES cases, with most reports indicating the presence of chronic inflammatory infiltrates associated with epithelial structures [4, 8, 9, 12], while a few cases have mentioned odontogenic epithelial proliferation [5, 12]. In the present case, histopathology of the soft tissues revealed features consistent with chronic inflammation, with the additional finding of subepithelial fibrous hyperplasia. Biochemical analyses remain limited, although Maki et al. [9] reported a calcium-to-phosphorus ratio (78.41%:21.59%) exceeding normal bone values, suggesting an active mineralization process rather than inert bone remnants.

### Clinical implications and diagnostic challenges

Eruption sequestrum typically occurs in children aged 6–12 years during the eruption of permanent teeth, most commonly affecting the first and second molars. Patients often present with mild chewing discomfort, accompanied by localized gingival swelling and erythema around the erupting tooth [9]. A firm, white-to-yellowish mass (usually **≤** 5 mm in diameter) may be palpated in the central fossa of erupting permanent teeth, and is occasionally observed at the distal marginal ridge. Its morphology is generally irregular, though it may correspond to the occlusal anatomy of the tooth [2, 7]. Radiography reveals a small, isolated, high-density bony fragment located coronal to the erupting tooth, positioned outside the dental follicle while maintaining an intact follicular space around the tooth, with no evidence of alveolar crest disruption or extensive bone destruction. Microscopic analysis demonstrates non-viable lamellar bone tissue devoid of osteoblastic or osteoclastic activity, accompanied by mild inflammatory cell infiltration (predominantly lymphocytes and macrophages) in the adjacent connective tissue. In a case reported by Watkins [2], histopathologic examination revealed the presence of bacteria within ES. Schuler et al. [7] associated ES with the accumulation of dental plaque biofilm, suggesting that prolonged exposure could lead to tooth demineralization or dental caries. Kennedy [4] observed inflammatory tissue in the surrounding soft tissues, proposing that ES may contribute to gingivitis, pericoronal inflammation, and even hinder the eruption of permanent molars. Furthermore, a case report by Ho [6] indicated that soft tissues containing embedded ES hard tissue fragments may undergo further organization, ultimately developing into fibromas.

## Discussion

The present case is exceptionally rare due to its simultaneous multiquadrant involvement (bilateral maxillary and mandibular first molars), challenging the traditional understanding of ES as a unifocal phenomenon, suggesting potential systemic or developmental predispositions. To date, ES has predominantly been reported in first permanent molars, which are the earliest erupting posterior teeth and are more susceptible to occlusal disturbances and pericoronal inflammation. There are currently no documented cases of ES involving premolars, which may be due to differences in eruption timing, crown morphology, or surrounding soft tissue anatomy. While rare or atypical presentations in premolars cannot be entirely excluded, available evidence suggests that ES is most commonly associated with molar eruption.

In this case, histopathologic examination revealed nonviable compact bone without evidence of odontogenic components such as cementum-like material, dentin, or pulp-like structures. These findings support the interpretation of the lesion as a sequestrum rather than a calcifying hamartoma, aligning more closely with the original theory proposed by Starkey and Shafer [1] whereby anatomically isolated cortical fragments evade resorption during eruption. Furthermore, the presence of chronic inflammation supports the hypothesis that local inflammatory responses may contribute to the sequestration process, as previously suggested by Roza et al. [12]. Notably, the sequential appearance of ES across multiple quadrants observed in this case presents a spatiotemporal pattern that cannot be easily explained by the random retention of devitalized bone fragments, nor does it represent true recurrence of previously affected sites. Instead, it suggests a pattern more consistent with developmental regulatory disturbances. The multiquadrant involvement in this patient raises the possibility of an underlying systemic or developmental predisposition, which warrants further investigation and may partially support the theory proposed by Onishi et al. [8]. Taken together with previous reports, this case suggests that the etiopathogenesis of ES may involve a multifactorial interplay of metabolic disturbances, chronic inflammatory responses, and developmental anomalies.

Although the histopathologic features observed in this case align more closely with those of osseous sequestration, we have carefully considered the developmental hamartoma hypothesis proposed by Onishi et al. [8] Classically, developmental hamartomas are characterized by the presence of mature dental tissues, including enamel, dentin, cementum, and pulp, arranged in either an organized or disorganized manner. These tissues are typically encapsulated by a fibrous connective tissue resembling the dental follicle, which demarcates the lesion from adjacent structures. However, none of these hallmark features were identified in our specimen. The lesion in this case was acellular, lacked evidence of neovascularization, and did not exhibit epithelial-mesenchymal interactions, all of which contrast distinctly with the known histological features of odontogenic hamartomas. For more definitive differentiation, immunohistochemical analysis utilizing specific markers such as cytokeratin 19, amelogenin, and dentin sialophosphoprotein may be considered to provide further evidence of tissue origin and differentiation.

The multiquadrant presentation of ES in this case posed unique diagnostic challenges requiring meticulous differential diagnosis. The diagnostic evaluation was primarily based on three key dimensions: (1) chronological correlation: the lesions demonstrated strict temporal correlation with tooth eruption phases, exhibiting progressive development synchronized with each molar’s emergence, which effectively distinguished them from static foreign body calcifications; (2) anatomical localization: their precise anatomical restriction to the occlusal surfaces of erupting molars contrasted sharply with the random jaw distribution of odontomas or the predictable positioning of residual roots in extraction sites; (3) radiographic characteristics: the lesions appeared as irregular radiopaque masses with enamel-like density, lacking the tooth-like structures typical of odontomas and the characteristic bony expansion or “honeycomb” radiolucency seen in calcifying epithelial odontogenic tumors (CEOT). Through this multidimensional assessment, we systematically excluded diagnoses such as CEOT, odontoma, traumatic residual roots, and foreign body reactions. Furthermore, histopathologic examination provided critical diagnostic confirmation, verifying the lesions as necrotic bone fragments rather than true odontogenic tumors. This comprehensive diagnostic approach—integrating chronological clinical features, anatomical distribution, radiographic morphology, and histopathological evidence - establishes a structured diagnostic framework for clinicians. Particularly noteworthy, the sequential quadrant involvement pattern holds dual significance: it effectively excludes traumatic etiologies while simultaneously informing optimal follow-up protocols and patient/parent counseling strategies. The observed spatiotemporal progression characteristics offer valuable insights for distinguishing developmental anomalies from acquired pathologies in clinical practice.

Despite the rarity of ES, characterizing its features is essential for updating histological knowledge and aiding clinicians in its diagnosis, management, and treatment. The 6-year-old girl in this case complained with pain due to food impaction, exhibiting significant indications for medical intervention. This rare case highlights the importance of proactive clinical surveillance in pediatric dentistry to prevent gingivitis, pericoronal inflammation or dental caries over time. Over long-term follow-up, this case was found to be exceptionally rare due to its simultaneous involvement of both the maxilla and mandible. The case was supported by comprehensive radiological evidence and histopathological evidence of both soft and hard tissues, which further refined our understanding of such rare conditions. It also contributes to the establishment of multicenter, evidence-based guidelines to determine the optimal intervention timing for both symptomatic and asymptomatic ES cases and to develop standardized management protocols. Future research employing immunohistochemical techniques may provide deeper insights into the role of osteoclast-osteoblast imbalance, epithelial-mesenchymal transition, and localized inflammatory dysregulation in the pathogenesis of ES.

## Conclusion

This rare multiquadrant ES case in the current report demonstrates the value of comprehensive evaluation (clinical, radiographic, and histopathological) for accurate diagnosis. The findings highlight: (1) the need for clinical awareness in pediatric dentistry, (2) importance of long-term follow-up for atypical eruptions, and (3) potential multifactorial etiology involving metabolic, inflammatory, and developmental factors. These insights provide a foundation for future research and guideline development.

## Data Availability

All data generated or analysed during this study are included in the manuscript and its supplementary information files. Due to the nature of this study (a case report containing potentially identifiable patient information), supporting clinical data cannot be made publicly available to protect patient privacy. De-identified data may be made available from the corresponding author on reasonable request, subject to approval by the institutional ethics committee and compliance with data protection regulations.

## Abbreviations

ES: Eruption sequestrum
CEOT: Calcifying epithelial odontogenic tumor

## References

[1] Starkey PE, Shafer WG. Eruption sequestra in children. J Dent Child. 1963;30:84–6.

[2] Watkins JJ. An unusual eruption sequestrum: a case report. Br Dent J. 1975;138:395–6. 1054987 10.1038/sj.bdj.4803465

[3] Gardner DE, Norwood JR. Eruption sequestra: five case reports. ASDC J Dent Child. 1978;45:475–6. 280555

[4] Kennedy DB. Eruption sequestrae: three case reviews. J Pedod. 1980;4:266–70.

[5] Priddy RW, Price C. The so-called eruption sequestrum. Oral Surg Oral Med Oral Pathol. 1984;58:321–6. 6592529 10.1016/0030-4220(84)90061-6

[6] Ho KH. Eruption sequestrum. Ann Acad Med Singap. 1986;15:454–5. 3777852

[7] Schuler JL, Camm JH, Houston G. Bilateral eruption sequestra: report of case. ASDC J Dent Child. 1992;59:70–2. 1537946

[8] Onishi T, Sakashita S, Ogawa T, Ooshima T. Histopathological characteristics of eruption mesenchymal calcified hamartoma: two case reports. J Oral Pathol Med. 2003;32(4):246–9. 12653866 10.1111/j.1365-2842.2004.01357.x-i1

[9] Maki K, Ansai T, Nishida I, Zhang M, Kojima Y, Takehara T, Kimura M. Eruption sequestrum: X-ray microanalysis and microscopic findings. J Clin Pediatr Dent. 2005;29:245–7. 15926442 10.17796/jcpd.29.3.477466743v232211

[10] Okawa R, Kimura KR, Fujita K, Nomura R, Nakano K, Fukuda Y, Ooshima T. Eruption sequestrum identified in mandibular molar region: case report and review of literature. Pediatr Dent. 2008;30:200–3.

[11] de Queiroz AM, Rocha CT, da Silva LA, Brentegani LG, da Silva RA, de Rossi A, Nelson-Filho P. Eruption sequestrum: case report and histopathological findings. Braz Dent J. 2012;23:764–7. 23338274 10.1590/s0103-64402012000600023

[12] Roza ALOC, de Abreu Brandi TC, Bezerra KT, Abrahão AC, Agostini M, de Andrade BAB, Vargas PA, Romañach MJ. Eruption sequestrum: an inflamed odontogenic hamartoma. Oral Maxillofac Surg. 2020;24:363–8. 32533408 10.1007/s10006-020-00865-4

